# Caffeic Acid Phenethyl Ester and Therapeutic Potentials

**DOI:** 10.1155/2014/145342

**Published:** 2014-05-29

**Authors:** Ghulam Murtaza, Sabiha Karim, Muhammad Rouf Akram, Shujaat Ali Khan, Saira Azhar, Amara Mumtaz, Muhammad Hassham Hassan Bin Asad

**Affiliations:** ^1^Department of Pharmacy, COMSATS Institute of Information Technology, Abbottabad 22060, Pakistan; ^2^University College of Pharmacy, University of Punjab, Lahore 54000, Pakistan; ^3^Department of Pharmacy, University of Sargodha, Sargodha 40100, Pakistan; ^4^Department of Chemistry, COMSATS Institute of Information Technology, Abbottabad 22060, Pakistan

## Abstract

Caffeic acid phenethyl ester (CAPE) is a bioactive compound of propolis extract. The literature search elaborates that CAPE possesses antimicrobial, antioxidant, anti-inflammatory, and cytotoxic properties. The principal objective of this review article is to sum up and critically assess the existing data about therapeutic effects of CAPE in different disorders. The findings elaborate that CAPE is a versatile therapeutically active polyphenol and an effective adjuvant of chemotherapy for enhancing therapeutic efficacy and diminishing chemotherapy-induced toxicities.

## 1. Introduction

**Figure 1:**
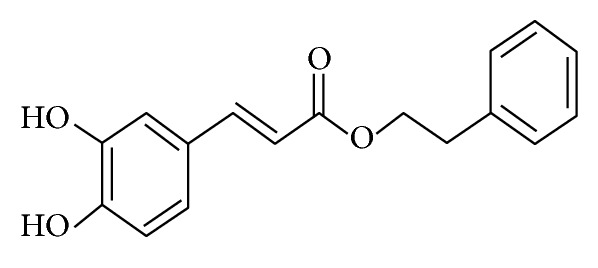
Chemical structure of caffeic acid phenethyl ester [3].

Caffeic acid phenethyl ester (CAPE) is a natural bioactive compound. It occurs in many plants [1]. It is acquired from propolis obtained through extraction from honeybee hives [2]. The chemical name of CAPE is 2-phenylethyl (2E)-3-(3,4-dihydroxyphenyl)acrylate. It is also termed as phenylethyl caffeate or phenethyl caffeate. Its molecular formula is C_17_H_16_O_4_ [3]. The chemical structure of CAPE is given in Figure 1. For the first time, Grunberger et al. identified this hydrophobic polyphenol [4]. This polyphenolic ester can also be synthesized by reacting caffeic acid with phenethyl alcohols [5–7]. CAPE is a polyphenol with hydroxyl groups within the catechol ring which is responsible for its crucial role in many biological activities [8]. The literature search showed an extensive research on the biological features of CAPE. The available studies narrate it as an effective moiety against various pathologies such as infections, oxidative stress, inflammation, cancer, diabetes, neurodegeneration, and anxiety [3, 5–8]. These therapeutic characteristics of CAPE have been summarized in this review article.

## 2. Activities of CAPE

Large number of studies has been conducted on various features of the biological and pharmacological activities of CAPE and its mode of action. Some of them are summarized below.

### 2.1. Antimicrobial Activity of CAPE

There are many studies which demonstrate the antimicrobial activity of CAPE against* Enterococcus faecalis*,* Listeria monocytogenes*,* Staphylococcus aureus* [9–11], and* Haemophilus influenzae* showing that RNA, DNA, and cellular proteins are possible targets of CAPE [9, 12]. Thus, dietary intake of CAPE is useful for the treatment of sore throat, common cold, and wound. There is evidence that CAPE possesses promising fungicidal activity on fungi infecting tomato without causing any harm to the fruit [13]. Moreover, poly(lactic-*co*-glycolic acid) (PLGA) sutures containing CAPE have been proposed to have antibacterial activity against* Staphylococcus aureus* and* Escherichia DH5*α** bacteria; this activity of CAPE was attributed to the synthesis of reactive oxygen species (ROS) that destroy the outer membrane of bacteria [14]. In recent studies [15–17], CAPE has been proposed as a valuable inhibitor of HIV-1 integrase; therefore, this polyphenol is believed to be a potential anti-HIV therapy. Fesen et al. reported that the integration step is efficiently inhibited by CAPE than the initial cleavage step by HIV-1 integrase [18]. In addition, CAPE and its esters, in a concentration range of 1.0 to 109.6 mM, have also been tested in an HCV replicon cell line of genotype 1b and found effective against replication of hepatitis C virus suggesting it a promising anti-HCV compound [19].

### 2.2. Anti-Inflammatory Activity

The anti-inflammatory activity of CAPE has also been documented [20, 21] (Table 1). The mode of anti-inflammatory activity of CAPE involves the inhibition of arachidonic acid release from the cell membrane; it, in return, inhibits the COX-1 and COX-2 activity as well as suppresses the activation of gene responsible for COX-2 expression [22–25]. In carrageenin-induced inflammation, CAPE suppresses both exudate volume and leukocytes relocation [26–28].

Moreover, the immunosuppressive behavior of CAPE has been evaluated in T-cells [29] because generally the causative agent for inflammation is T-cells [25]. This discovery revealed the CAPE-mediated inhibition of initial and late steps in T-cell receptor-mediated T-cell activation [28] and thus proposed the mechanistic basis for the immunomodulatory and anti-inflammatory activities of CAPE. Furthermore, inhibition of both interleukin- (IL-) 2 gene transcription and the IL-2 synthesis by CAPE in stimulated T-cells was also observed. In Jurkat cells, binding with DNA and the activities of transcription factors, such as NF-*κ*B, nuclear factor of activated cells (NFAT), and activator protein-1 (AP-1) were also characterized to examine the mode of inhibition of the transcription phase by CAPE [29–32]. The results elaborated the CAPE-mediated inhibition of NF-*κ*B-dependent transcription showing no effect on the disposition of IB*κ*B (cytoplasmic NF-*κ*B inhibitory protein). In CAPE-treated Jurkat cells, there was restricted binding of NF-*κ*B to DNA and transcriptional activity of a Gal4-p65 hybrid protein. In addition, CAPE-mediated inhibition of binding with DNA and transcriptional activity of NFAT in CAPE-treated Jurkat cells was also seen [29].

For the assessment of CAPE effects, Sanghyum and Seok-Jai used human neutrophils activated by lipopolysaccharide [33]. The results of this study elaborated the CAPE-mediated inhibition of the production of TNF-*α* and IL-6 factors. Moreover, the attenuation of phosphorylation potentials of ERK1/2 and JNK was also observed. As a conclusion, these outcomes show the potential usage of CAPE for controlling inflammation caused by neutrophils.

In CAPE-treated gastric epithelial cell line (AGS), an obstruction was observed in cytokine- and mitogen-provoked NF-*κ*B and AP-1 expression [34]. Additionally, CAPE inhibited the* H. pylori*-provoked cell proliferation,* H. pylori*-induced COX-2 expression, and synthesis of the cytokines, TNF-a, and IL-8. These results are potential insights into the anticancer and anti-inflammatory activities of CAPE.

### 2.3. Cytotoxicity of CAPE

An extensive literature is available regarding cytotoxicity studies of CAPE as documented in Tables 1 and 2. In the presence of CAPE, human pancreatic and colon cancer cells undergo apoptosis [35, 36]. The in vitro and in vivo studies reveal the growth inhibition of C6 glioma cells by CAPE [37].

There are many evidences [38–42] which elaborate the antiproliferation activity of CAPE. For normal cellular proliferation, adequate levels of nuclear factor (NF)-*κ*B activity must be maintained. In some cancers, elevated activation of NF-*κ*B is observed. To obstruct the NF-*κ*B activation phenomenon, CAPE has been proved to be effective chemopreventive agent [38, 42]. Nutritional ingestion of CAPE may thus be valuable for patients whose tumors express steadily elevated levels of activated NF-*κ*B, for instance, squamous head and neck carcinomas. It has been reported that CAPE render antitumor features [43] devoid of causing cytotoxicity to normal cells [44]. Su et al. proposed that cytotoxicity of CAPE is directly related to its apoptotic effect [45].

The antitumor activity of CAPE has been investigated to reveal its influence on cancer development including angiogenesis, tumor invasion, and metastasis. Liao et al. carried out a cytotoxicity study of CAPE in colon adenocarcinoma cells (CT26) and reported a dose-dependent decline in cell viability [46]. Moreover, there was reduction in both expression of matrix metalloproteinase and production of vascular endothelial growth factor from CAPE-treated CT26 cells resulting in the reduced angiogenesis and metastasis [47]. These observations provide insight into the promising antimetastatic feature of CAPE. In addition, Song et al. reported that antiangiogenic property of CAPE was also accounted for its anti-inflammatory effect because angiogenesis and chronic inflammation depend on each other [48]. An anti-inflammatory response is obtained by blockage of angiogenesis [49–51]. As far as mode of anticancer activity of CAPE is concerned, CAPE is capable of (i) inhibiting the xanthine oxidase which can metabolize both purine and pyrimidine bases and obstruct the nucleotide production pathway [52–54]; (ii) suppressing 5-lipoxygenase [55]; (iii) inhibiting the tumor promoter-mediated oxidative responses in the culture of HeLa cells [56]; (iv) inhibiting the azoxymethane-provoked colonic preneoplastic lesions and enzymatic processes related to colon carcinogenesis [44]; (v) inducing apoptosis [57]; and (vi) modulating the redox state of the cells [51, 58–60].

### 2.4. CAPE against Chemotherapy- and Irradiation-Induced Toxicities

Due to free radical formation and oxidant injury, many drugs used for the treatment of cancer destroy the physiological homeostasis of many organs, such as kidneys and liver [61, 62]. It reduces therapeutic efficacy of anticancer drugs and produces undesired effects, such as doxorubicin, and cisplatin causes nephrotoxicity, while tamoxifen produces hepatotoxicity. Methotrexate activates the NF-*κ*B which causes mucosal barrier injury. These side effects limit their use as anticancer chemotherapy.  Table 3 lists various studies conducted to explore the role of CAPE in minimizing undesired effects of some anticancer drugs, that is, doxorubicin, cisplatin, methotrexate, bleomycin, and tamoxifen. The usage of CAPE suppresses free radical formation [63]; thus chemotherapy-induced toxicities are diminished. Moreover, CAPE inhibits the NF-*κ*B factor which amplifies the susceptibility of intestinal epithelial cells to anticancer drugs, preferably methotrexate [64, 65]. So, these studies present CAPE to be an effective adjuvant of chemotherapy for enhancing therapeutic efficacy and diminishing chemotherapy-induced toxicities.

The usage of CAPE against irradiation-induced toxicities of tumor cells has also been done and very promising results are seen (Table 4). Many genes are affected by transcription factor NF-*κ*B resulting in various disorders, such as immune and inflammatory syndromes. The activation of NF-*κ*B induced by irradiation stimulates various undesired effects, such as an inflammatory response in the intestines. Since CAPE has emerged as an effective inhibitor of NF-*κ*B, CAPE has been applied in different animal models and cell lines to understand inflammatory phases after irradiation [66]. Table 4 enlists various studies which suggest that CAPE can promisingly avert the development of postirradiation inflammation.

For successful radiotherapy, the application of CAPE in radiosensitization of tumor cells has been studied (Table 5). By ionizing radiation, the increased death of CAPE treated cells has been reported [67]. Since CAPE is an effective inhibitor of NF-*κ*B and a stimulator of the functions of glutathione S-transferase, it drains GSH levels. Subsequently, tumor cells are radiosensitized due to this drainage.

### 2.5. Miscellaneous Activities of CAPE

The studies have demonstrated that CAPE possesses neuroprotective activity [68–71]. CAPE can obstruct apoptosis in cerebellar granule cells [72], reduce ischemia/reperfusion-provoked cerebral injury [73, 74] and spinal cord ischemia/reperfusion injury [75], and avert different toxin-provoked neurotoxicity [69, 76–79]. Moreover, CAPE efficiently suppresses NF*κ*B activation [55], lipid peroxidation activity [38], lipoxygenase activities [80], protein tyrosine kinase activity [81], and ornithine decarboxylase activity [82].

As a result of cellular metabolism, reactive oxygen species (ROS) are generated. The representative examples of ROS include hydrogen peroxide (H_2_O_2_), the superoxide anion, hydroxyl ion, and reactive nitrogen species, especially nitric oxide [83]. The enzymatic processes usually detoxify these ROS [84]. On the other hand, ROS build up if an imbalance is established between their synthesis and degradation resulting in oxidative stress. The excessive ROS can react to the macromolecules such as DNA resulting in some harmful responses [85]. Large research has been done to assess antioxidant role of CAPE [10, 86]. The evidences show that CAPE is potent antioxidant which can scavenge ROS and protect the cell membrane against lipid peroxidation [87–91]. Some other studies elaborate immunomodulator [4, 38, 92], antihepatotoxic [93], antiosteogenic [94], and antiatherosclerotic [95] (Table 1) role of CAPE. Moreover, Ucan et al. have proposed the effect of CAPE on bone healing in a rat model [96].

## 3. Conclusion

Considering the preceding literature, CAPE can be suggested to possess various activities, such as antimicrobial, antioxidant, anti-inflammatory, and cytotoxicity activities. The advance investigations are required about the clinical prospective toxicities of CAPE if it is going to be used as a therapeutic agent. Extensive literature is available on some research areas, but still some areas are very much less or not explored; therefore, further investigations to use this common and economic polyphenol for the best therapeutic treatment of ailments are required and proposed.

## Figures and Tables

**Table 1:** Various activities and molecular targets of CAPE.

Number	Activity	Molecular targets of CAPE	References
1	Antioxidant	ROS	[22, 55, 86, 97–103]
2	Anti-inflammatory	COX-1, COX-2, NF-*κ*B, NFAT, and AP-1	[4, 23, 38, 104–106]
3	Anticarcinogenic	NF-*κ*B	[4, 38, 92, 107, 108]
4	Antiviral	HIV-1 integrase	[55]
5	Immunomodulator	NF-*κ*B	[4, 109]
6	Antihepatotoxic	CYP2El	[93]
7	Neuroprotective	ROS	[110]
8	Antiatherosclerotic	NF-*κ*B	[95]

**Table 2:** Cytotoxicity studies of CAPE in different cells.

Number	Cells	References
1	Human pancreatic cancer cells	[35]
2	Human colon cancer cells	[36]
3	C6 glioma cells	[37]

**Table 3:** Applications of caffeic acid phenethyl ester against chemotherapy-induced toxicities.

Number	Toxicity inducing drugs	Toxicity	Subject	References
1	Doxorubicin toxicity	Nephrotoxicity	Rats	[111]
2		Cardiotoxicity	Rats	[112]
3		Neuronal oxidant injury	Rats	[113]
4		Medulloblastoma cell toxicity	Human	[114]

5	Cisplatin toxicity	Nephrotoxicity	Rats	[63]
6		Bone marrow cell toxicity	Rats	[115]
7		Hepatotoxicity	Rats	[116]
8		Hepatotoxicity	Rats	[117]
9		Ototoxicity	Rats	[114]

10	Methotrexate toxicity	Neuronal oxidant injury	Human	[118]
11		Nephrotoxicity	Rats	[119]
12		Cerebellar oxidative stress	Rats	[120]
13		Testicular toxicity	Rats	[121]
14		Nephrotoxicity	Rats	[122]
15		Hepatorenal oxidative injury	Rats	[123]
16		Hepatorenal oxidative injury	Rats	[124]

17	Bleomycin toxicity	Lung fibrosis	Rats	[124, 125]

18	Tamoxifen toxicity	Hepatotoxicity	Rats	[126]

**Table 4:** Applications of caffeic acid phenethyl ester against chemotherapy-induced toxicities of tumor cells.

Number	Toxicity	Subject	References
1	Lung injury	Rats	[127]
2	Medulloblastoma cell toxicity	Rats	[128]
3	Ileal mucosal toxicity	Rats	[75]
4	Normal lung fibroblast and lung cancer cell line	Rats	[129]

**Table 5:** Applications of caffeic acid phenethyl ester in radiosensitization of tumor cells.

Number	Cell lines used	References
1	Colorectal adenocarcinomas (CT26)	[67]
2	Lung cancer cells (A549) and normal lung fibroblast cells (WI-38)	[130]

## Abbreviations

CAPE: Caffeic acid phenethyl ester
ROS: Reactive oxygen species
HIV: Human immunodeficiency virus
HCV: Hepatitis C virus
COX: Cyclooxygenase
IL: Interleukin
NF-*κ*B: Nuclear factor kappa-light-chain-enhancer of activated B cells
NFAT: Nuclear factor of activated cells
AP-1: Activator protein-1
IB*κ*B: Cytoplasmic NF-*κ*B inhibitory protein
ERK: Extracellular signal-regulated protein kinases
JNK: c-Jun NH_2_-terminal kinase
TNF: Tumor necrosis factor
GSH: Glutathione
H_2_O_2_: Hydrogen peroxide.
